# Methods for a similarity measure for clinical attributes based on survival data analysis

**DOI:** 10.1186/s12911-019-0917-6

**Published:** 2019-10-21

**Authors:** Christian Karmen, Matthias Gietzelt, Petra Knaup-Gregori, Matthias Ganzinger

**Affiliations:** 1 Heidelberg University Hospital, Institute of Medical Biometry and Informatics, Im Neuenheimer Feld 130.3, 69120 Heidelberg, Germany; 2 Peter L. Reichertz Institute for Medical Informatics of TU Braunschweig and Hannover Medical School, Carl-Neuberg-Str. 1, 30625 Hannover, Germany

**Keywords:** Case-based reasoning, Similarity measure, Survival data, Clinical decision support

## Abstract

**Background:**

Case-based reasoning is a proven method that relies on learned cases from the past for decision support of a new case. The accuracy of such a system depends on the applied similarity measure, which quantifies the similarity between two cases. This work proposes a collection of methods for similarity measures especially for comparison of clinical cases based on survival data, as they are available for example from clinical trials.

**Methods:**

Our approach is intended to be used in scenarios, where it is of interest to use longitudinal data, such as survival data, for a case-based reasoning approach. This might be especially important, where uncertainty about the ideal therapy decision exists. The collection of methods consists of definitions of the local similarity of nominal as well as numeric attributes, a calculation of attribute weights, a feature selection method and finally a global similarity measure. All of them use survival time (consisting of survival status and overall survival) as a reference of similarity. As a baseline, we calculate a survival function for each value of any given clinical attribute.

**Results:**

We define the similarity between values of the same attribute by putting the estimated survival functions in relation to each other. Finally, we quantify the similarity by determining the area between corresponding curves of survival functions. The proposed global similarity measure is designed especially for cases from randomized clinical trials or other collections of clinical data with survival information. Overall survival can be considered as an eligible and alternative solution for similarity calculations. It is especially useful, when similarity measures that depend on the classic solution-describing attribute “applied therapy” are not applicable. This is often the case for data from clinical trials containing randomized arms.

**Conclusions:**

In silico evaluation scenarios showed that the mean accuracy of biomarker detection in k = 10 most similar cases is higher (0.909–0.998) than for competing similarity measures, such as Heterogeneous Euclidian-Overlap Metric (0.657–0.831) and Discretized Value Difference Metric (0.535–0.671). The weight calculation method showed a more than six times (6.59–6.95) higher weight for biomarker attributes over non-biomarker attributes. These results suggest that the similarity measure described here is suitable for applications based on survival data.

## Background

### Introduction

Solving problems on the basis of a solution that worked for a similar problem in the past is a well-known human strategy. In the field of medicine, this principle is applied either knowingly or unknowingly when a physician recalls past cases and how they were treated. Modelling this approach into computer systems has been subject of research for decades. For example, the case-based reasoning (CBR) methodology has been developed from the 1980s onwards [1, 2]. It has been applied, for example, to electronic health records as a secondary use [3, 4]. More recently, patient similarity has been recognized as an important principle for systems medicine and precision medicine [5]. Since it is a broad and general approach, CBR and the underlying similarity measures can be applied to a variety of fields. Successful models and clinical decision support systems were first applied for medical fields like dentistry [6], osteopathy [7], psychology [8], diabetes [9], and other complex diseases like cancers [10–12].

For use in an electronic decision support system, a case base is established to provide historic case descriptions and solution approaches. Each case is described by a set of attributes such as symptom descriptions or laboratory values, treatments, and the outcome. To quantify similarity between two cases, for each of these attributes a similarity measure has to be defined that will provide a local similarity value for the two instances of the attribute. A variety of similarity measures has been described, for example by fuzzy matching [13], cross-correlation [14], and Bayes’ theorem [15]. For this paper, it is important to distinguish between numeric and nominal value domains of attributes. If an attribute has a numeric value domain, like body temperature, the similarity between two instances can be calculated by a function like the Euclidian distance function. In contrast, for attributes with a nominal value domain like blood groups, it is often necessary to prepare a context-specific matrix representing the similarity values of all possible value pairs.

In many similarity measures, the overall or global similarity between two cases is achieved by accumulating these local similarity values into a single similarity value. The difficulty here is to decide how much impact a single attribute has on the overall similarity (attribute’s weight). For example, the attribute “sex” might be less relevant for overall similarity in a specific context like sepsis than the attribute “fever”. In this example, the weight of “fever” should be higher than the weight of “sex”.

The local similarity matrices of nominal attributes and the weights of attributes are often defined manually by domain experts like medical specialists [8, 16–18]. This works well for straightforward domains with low complexity. However, in complex domains even clinical experts in the same field may have different views on the impact of an attribute on the disease of interest. A more objective approach is to derive the similarity from the data in the case base. There are a number of CBR algorithms that are able to learn local similarities from the case base itself. However, many of them are based on the dependency of one or more solution-describing attributes. In clinical contexts, this is often the case for the attribute “applied therapy”. A special case where such CBR based systems struggle is when data from randomized clinical trials is analyzed. Here, this dependency would cause a huge bias, because therapy arms (novel therapy against gold standard or placebo therapy) are usually randomized. As an alternative, a similarity measure depending on “overall survival time” might make more sense as it is considered authentic in assessing the success of clinical trials. However, the authors of this article are not aware of the existence of a similarity measure with an explicit focus on “overall survival time”.

A problem in obtaining knowledge from clinical or laboratory data is that the influence of each attribute on the given disease might not be fully discovered yet. As a consequence, for many of the complex diseases mentioned above it is not always clear, which therapy is the individually most suitable for a given patient. Usually, the therapy that showed the best overall performance for a patient cohort is recommended for all patients. This, however, neglects the possibility that subtypes with uneven distribution might exist, where patients with a rare subtype might benefit more from a non-standard treatment. To address these issues, we propose several methods for a similarity measure that are based on the analysis of survival data as they are available for example from clinical trials. Each method may be used independently from the others. Additionally, we propose a completely composed measure as an example. For patients suffering from life-threatening diseases like cancers, the outcome *survival time* is often considered the most important measure for the therapy success. In contrast to existing solutions, we calculate the similarity matrix based on the survival probability that is associated with the values of an attribute.

For our approach, we analyze survival data of patients in our case base with the help of survival functions. Consequently, we learn the significance of each case-describing attribute with respect to survival time. Weights for merging the local similarity values into a global value are calculated on the basis of survival data as well. The attributes describing a case may include all types of structured clinical data, because both, numeric and nominal values can be processed. The resulting similarity measure is designed for easy integration into CBR frameworks, such as myCBR [19, 20] and eXiT*CBR [21].

### Related work

In the last years, many new approaches have been developed in the field of CBR and related topics such as similarity measures and information retrieval. For example, Goel and Diaz-Agudo provide a comprehensive overview on the development in the field [22]. Especially interesting examples are works on textual CBR and spatial CBR. Textual CBR is a subdomain of CBR where the knowledge source is available in textual form. In the clinical domain, this could be medical reports, like discharge or referral letters. In order to retrieve knowledge from unstructured text data, further techniques must be applied initially to transformation information into structured case representations [23]. A common way to achieve this is the textual analysis with methods from natural language processing [24]. An example for spatial CBR is Q-CBR (Qualitative Case-Based Reasoning) that has shown promising results using Qualitative Spatial Reasoning (QSR) theory for retrieval in the technical domain of robotics artificial intelligence [25]. Here, qualitative spatial relations between objects are assumed, aiming to model the human common sense understanding of space.

Closely related to similarity measures, distance functions are often used to determine differences in an absolute vector space. So, instead of a similarity that usually has a value range of [0.0, 1.0], a distance function between two attributes may result in any decimal number. However, a conversion from a distance function to a similarity function is feasible in many cases. The by far most commonly used methods are the Euclidian Distance function and the Manhattan (city-block) function. Both are equivalent to the Minkowskian r-distance function [26] with *r* = 1 and 2, respectively, however, they do not handle non-numeric (nominal) attributes appropriately.

The Heterogeneous Euclidian-Overlap Metric (HEOM) [27, 28] tackles this issue by a dedicated handling of nominal and continuous attributes. The overlap metric applies for nominal attributes and results in a distance of 1.0 for matching and 0.0 for not matching attributes, respectively. On the contrary, for linear attributes the numeric value difference of the attributes is normalized by dividing by the range of all possible values for that specific attribute a (range_a_ = max_a_-min_a_). The normalization fails, however, if the value range is defined too tight. Also, the nominal value handling is not able to compute distances other than the extreme ones. Expert domain knowledge must be added to further differentiate such cases.

The Value Difference Metric (VDM) [29] was initially introduced by Stanfill and Walz. In this approach the difference between two nominal values (of the same attribute) depends on the conditional probability that the output class is c, given that attribute a has the value x: P(c|x_a_). Wilson and Martinez [30] published an improved version of VDM that adds the ability to handle continuous attributes. This is done by transforming them into a fixed number of equally sized intervals that enables them to be treated in the same way as a nominal attribute (DVDM, short for Discretized VDM). The overall distance of two cases is then determined by the Euclidian Distance. The Interpolated and Windowed VDM (IVDM/WVDM) are furthermore smoothing the steps between probability input classes. The VDM‘s strength is the assignment of case bases with verified knowledge about the solution that is known to be the best available. However, it cannot learn local similarities when the solution attribute is numeric, like the overall survival.

## Methods

In a typical randomized controlled trial a new therapy (e.g. medication) is compared to either the standard or a placebo medication (competing therapy). For fatal diseases, like many cancer types, the performance between the new and the competing therapy is compared with the help of overall survival (OS) information of each therapy group (sub-cohort) after therapy onset. The survival function basically represents the probability of survival over time.

As a result, survival functions enable the visualization of the survival probability (y-axis) over time (x-axis). This method is commonly used to compare the outcomes of two competing therapies in clinical trials. When two therapies are compared, the one with predominantly higher survival probabilities is considered superior since subjects tend to survive or to die later in the course of observation.

### Survival functions as a measure for similarity

In evaluations of clinical trials, survival functions are calculated and plotted to visually represent the difference in survival for each attribute value, e.g. for each therapy arm of the study cohort. This way the differences in survival probabilities of the study arms can be visually compared and also calculated for any point in time. As a result, an extensive survival analysis on attribute value level can be performed and will be used to define local similarities as a consequence.

Formally, survival functions are defined as follows: Let *f* be a probability density function. Then, the survival function *S* : [0, ∞) → [0, 1] depending on time *t* is defined as

1 $$ S(t)=\underset{t}{\overset{\infty }{\int }}f\left(\tau \right)\  d\tau . $$

This means that the survival probability at time *t* = 0 starts with *S*(*t* = 0) = 1 and decreases over time. Thereby, *S*(*t*) is bound to the interval [0, 1].

**Fig. 1:**
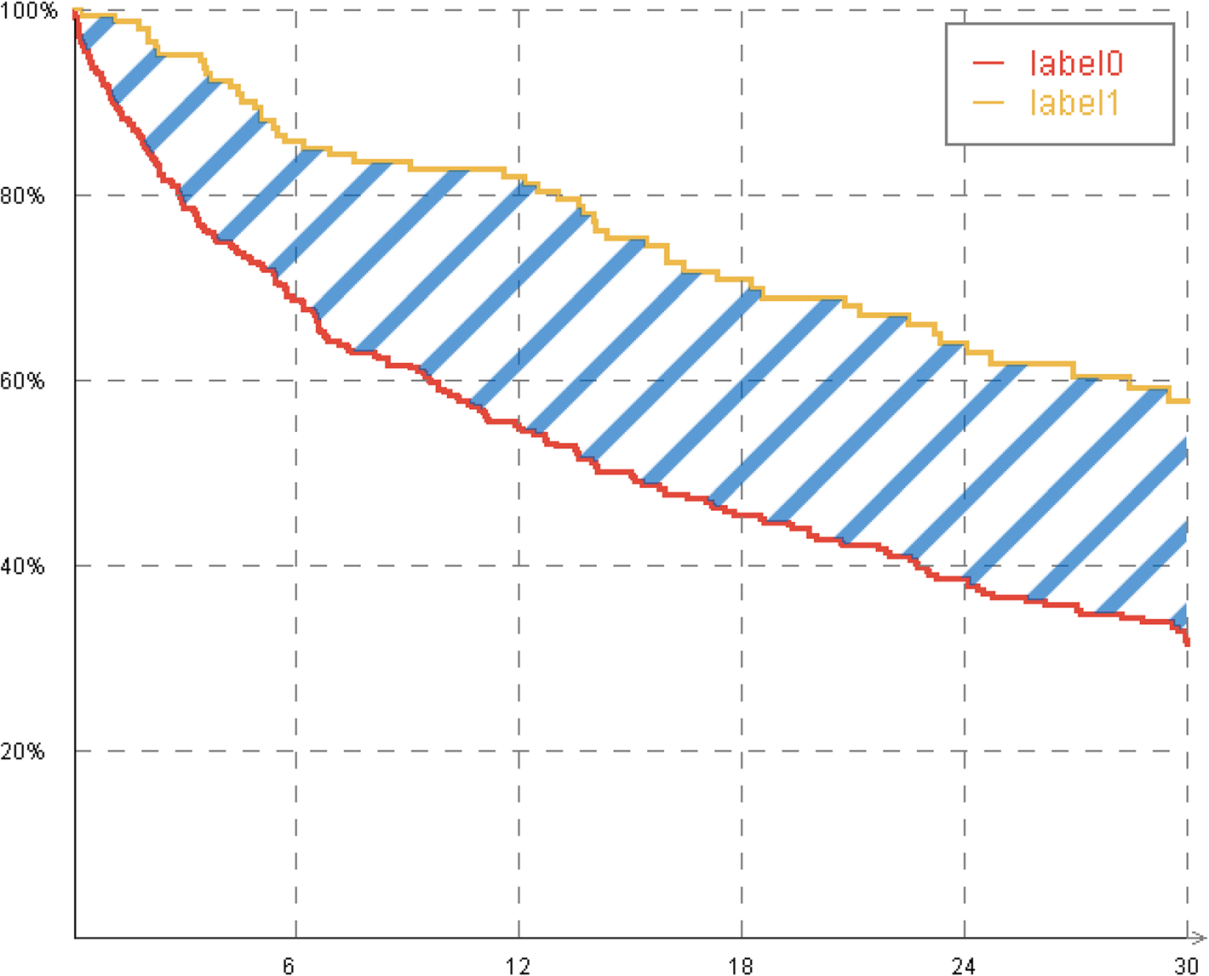
Survival plots of two values “label0” and “label1” of an attribute are shown. The shaded area between these plots is the Area Between Survival functions (ABS)

In our approach, the Area Between two Survival functions (ABS, as shown in Fig. 1) is considered a measure for the similarity of two values of the same attribute. Details for calculating the ABS are presented in the next section. The following two examples demonstrate possible scenarios for similarities:

- Scenario 1: Marginal differences between two survival functions will occur when two sub-cohorts are compared with respect to an unimportant attribute. For example, in a cancer therapy group where the survival probabilities are almost equal for the attribute “sex” with its values “male” and “female”. Here, the ABS between both cohorts is very small.
- Scenario 2: Huge differences are expected when comparing two highly discriminating values of a single attribute with regard to survival. The attribute “metastasis formation” with the values “none” and “end stage”, for example, will probably have an extreme impact on the survival probability in cancer therapy. Here, the sub-cohort with “none” metastasis will have a better survival outcome than the “end stage” group. This leads to a relatively high ABS.

### Formal notations and definition

Let *C* = {*C*_1_, *C*_2_, …, *C*_*m*_} be the set of all cases in the case base and *A* = {*A*_1_, *A*_2_, …, *A*_*n*_} be the set of all attributes. Let *a* ∈ *A*_1_ × *A*_2_ × … × *A*_*n*_ be an attribute vector of a certain case and let ID be the set of unique case IDs, i.e. *y*_*i*_, *y*_*j*_ ∈ ID satisfy *y*_*i*_ = *y*_*j*_ ⇔ *i* = *j* ∀ *i*, *j*. Then, a certain case *c* ∈ *C* is defined as a tuple of *c* = (*y* ∈ ID, *a*).

Let *c*, *c*^∗^ be two cases of the case base and *c* = (*x*, *a*) and *c*^∗^ = (*x*^∗^, *a*^∗^) with IDs *x*, *x*^∗^ ∈ ID and attribute vectors *a*, *a*^∗^ ∈ *A*_1_ × *A*_2_ × … × *A*_*n*_. *a*, *a*^∗^ are defined as *a* = (*a*_1_, *a*_2_, …, *a*_*n*_) and *a*^∗^ = (*a*_1_^∗^, *a*_2_^∗^, …, *a*_*n*_^∗^).

The attribute values *a*_*i*_ of *a* and *a*_*i*_^∗^ of *a*^∗^ with *i* = {1, 2, …, *n*} of the two cases *c* and *c*^∗^ can only be compared pairwise for similarity. The Area Between two Survival functions (ABS) of a particular pair *i* of attribute values and a particular point in time *T* can be defined as:

2 $$ \mathrm{ABS}\left(T|{a}_i,{a_i}^{\ast}\right)=\underset{0}{\overset{T}{\int }}\left[S\left(t|{a}_i\right)-S\left(t|{a_i}^{\ast}\right)\right]\ \mathrm{d}t $$

**Fig. 2:**
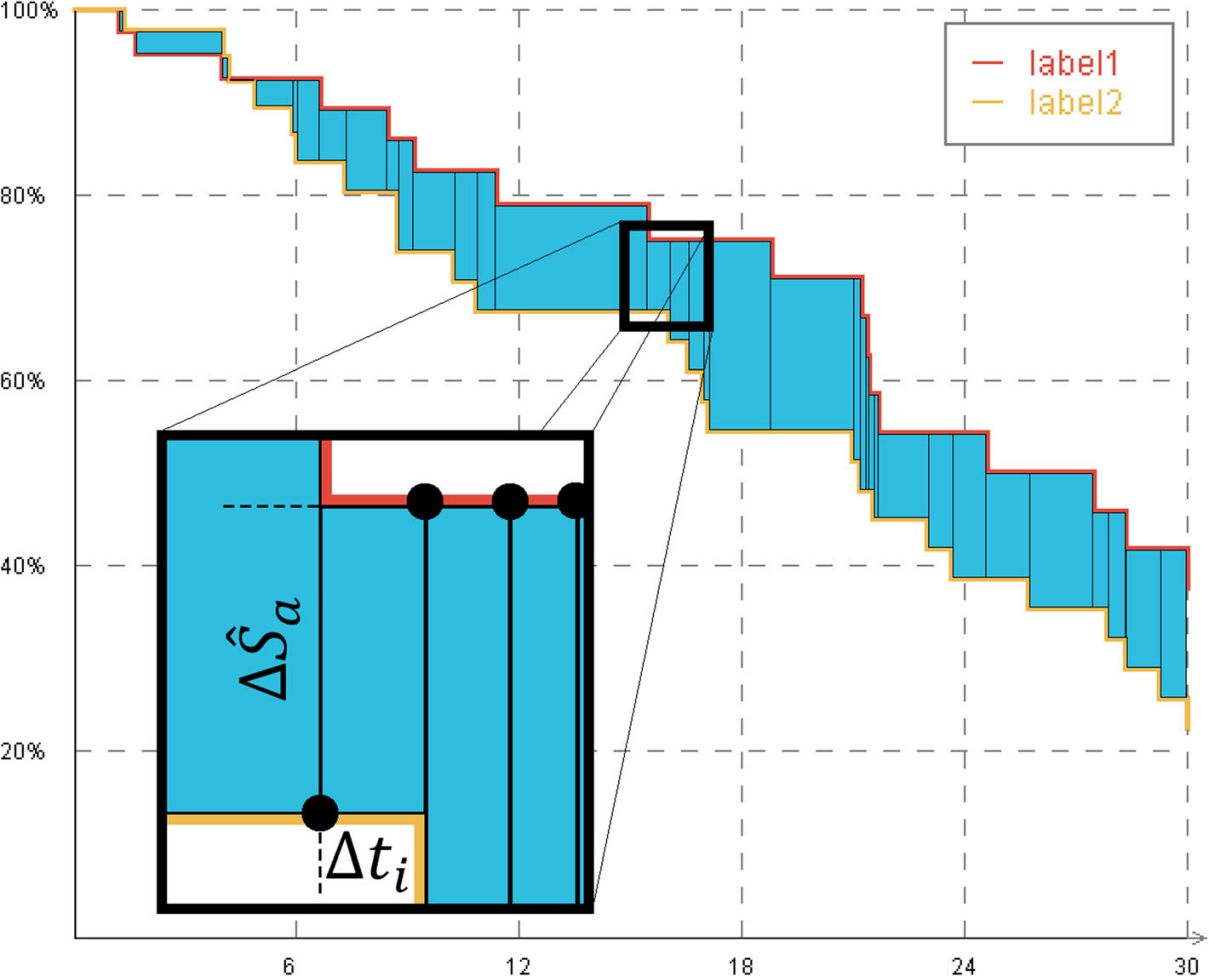
Rectangles for the calculation of the Area Between Survival functions (ABS)

We consider the survival function as a polygonal function between the data points resulting in a step function (cf. Figure 2). Now, the ABS between two succeeding events can be considered as a rectangle. The calculation of the complete ABS can now be achieved by summing up all single rectangles. Please note that the ABS can only be calculated for two values *a*_*i*_ and *a*_*i*_^∗^ of the identical attribute *A*_*j*_.

### Similarity metrics

The following subsections cover suggested transfer methods from our survival-data-based similarity concept to the different parts of a similarity measure. Furthermore, in section 2.3.4 we combine some of these methods to a complete similarity metric that can be applied in CBR.

#### Local similarity

Like many other similarity measures [31, 32] and CBR frameworks [21], we adopted the concept of differentiating between a local and a global similarity function and applied it to our approach. The local and global similarity measures are usually defined within the interval [0, 1]. Consequently, the ABS has to be transformed to meet this constraint. Since there are numerous transformations available, we propose the use of the following:

3 $$ {\mathrm{sim}}_{local}\left(T|{a}_i,{a_i}^{\ast}\right)=\exp \left(-\left|\mathrm{ABS}\left(T|{a}_i,{a_i}^{\ast}\right)\right|\right) $$

Since the similarity of two attribute values with a low absolute value of ABS is higher compared to attribute values with a large difference in the survival functions, we map the ABS using an exponential function. This limits the local similarity to the interval [0, 1] and provides low local similarity values for high ABS values and a maximum similarity of 1, if the attribute values are the same.

#### Attribute weights and feature selection

In our approach, the purpose of the attributes’ weights is to define the survival impact of each attribute on a global scale. We define this global scale with the help of a special normalization area ABS_norm_ that reflects the attribute with the most extreme impact with reference to survival: the survival status.

4 $$ {\mathrm{ABS}}_{\mathrm{norm}}(T)=\underset{0}{\overset{T}{\int }}\left[S\left(t|\mathrm{alive}\right)-S\left(t|\mathrm{deceased}\right)\right]\ \mathrm{d}t $$

**Fig. 3:**
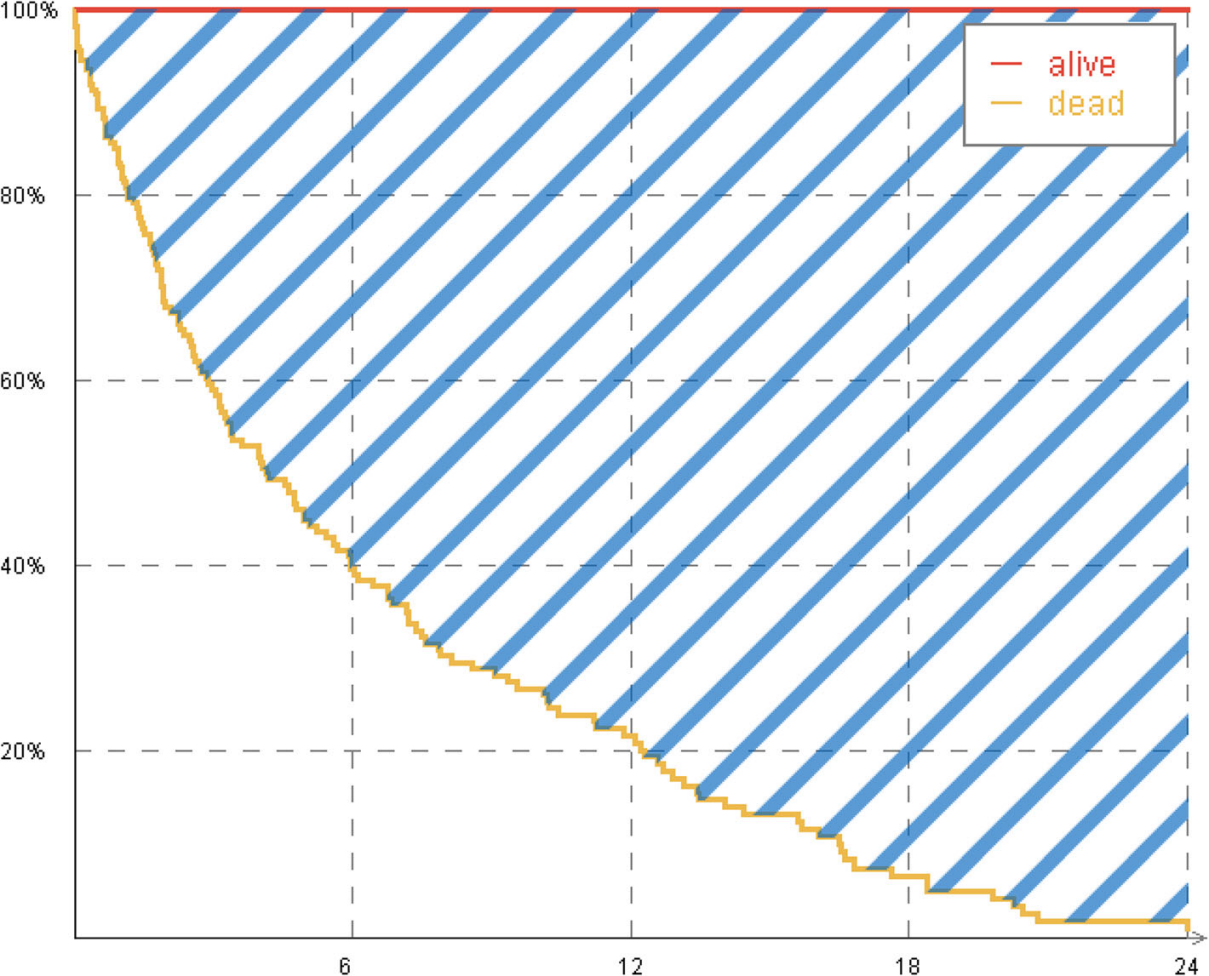
ABS of attribute “survival status”: *ABS*_*norm*_

No other attribute can possibly have values with a bigger impact on survival than the survival status itself. The resulting ABS_norm_ is illustrated in Fig. 3 as striped area.

**Fig. 4:**
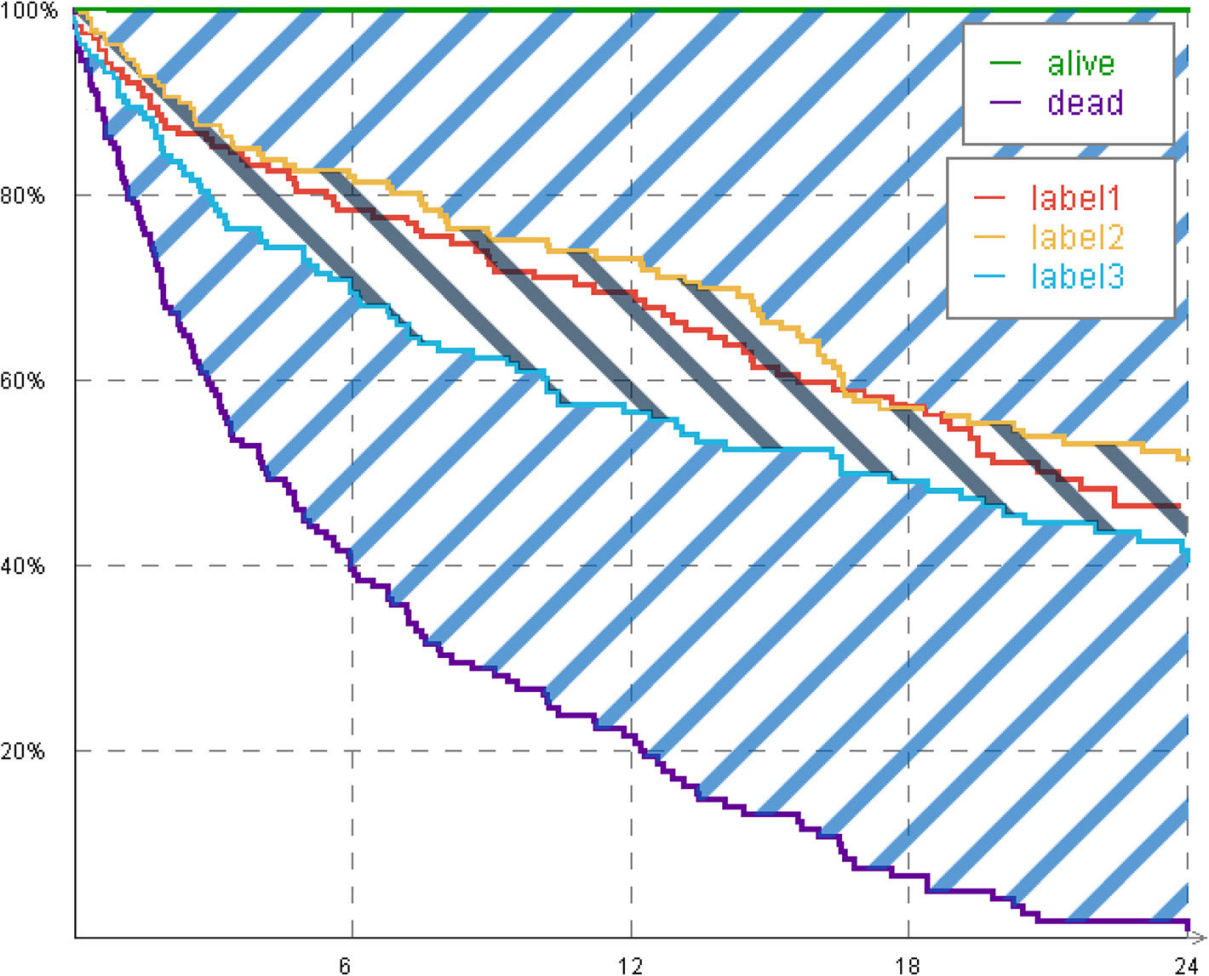
Comparison between ${ABS}_{\mathit{\max},{A}_j}$ (area between label2 and label3) and *ABS*_*norm*_ (same area as in Fig. 3) used for calculation of an attribute’s weight on a global scale

In order to determine the impact of a particular attribute *A*_*j*_ on a global scale, the maximum ABS of the attribute is computed$\left({\mathrm{ABS}}_{\max, {A}_j}\right).$This area provides information about how much impact the attribute has with respect to all other attributes. The attribute’s weight ${\omega}_{A_j}$ is the rate between area ${\mathrm{ABS}}_{\max, {A}_j}$ and the normalization area ABS_norm_ as in Eq. (5). Figure 4 shows a visual comparison between both areas.

5 $$ {\omega}_{A_{\mathrm{j}}}=\frac{{\mathrm{ABS}}_{\max, {A}_j}}{{\mathrm{ABS}}_{\mathrm{norm}}} $$

The concept of using weights is furthermore particularly suitable for feature selection, because attributes with a low impact on survival will get a correspondingly low weight. Attributes below a certain weight’s threshold could be omitted to reduce overall computation complexity without decreasing the accuracy of similarities.

#### Handling of numeric attributes

A characteristic of survival estimators is that they are limited to nominal attribute values. Clinical data, however, usually contains a large portion of attributes with numeric values, e.g. laboratory results or other measurements that are considered relevant for diagnosis or therapy.

For continuous value domains, survival estimators could be applied by interpreting each number as a nominal value, but this approach would lead to an extreme overfitting. Especially decimal values, like a specific laboratory attribute, are rarely repeated and thus could lead to as many survival functions as there are cases in the case base. To cope with this issue, numeric attributes have to be nominalized. The goal is to transform the attribute’s numeric range into two nominal groups. A cutoff value for dichotomization is chosen that maximizes the ABS between both groups.

As a first step, a temporary cutoff value *c* is set for each unique value of the numeric attribute to normalize. For a temporary cutoff value *c* the ABS between the two groups “less than or equal to *c* ” and “greater than *c* ” is calculated:

6 $$ \mathrm{ABS}\left(T|{a}_i\le c,{a}_i>c\right)=\underset{0}{\overset{T}{\int }}\left[S\left(t|{a}_i\le c\right)-S\left(t|{a}_i>c\right)\right]\ \mathrm{d}t $$

**Fig. 5:**
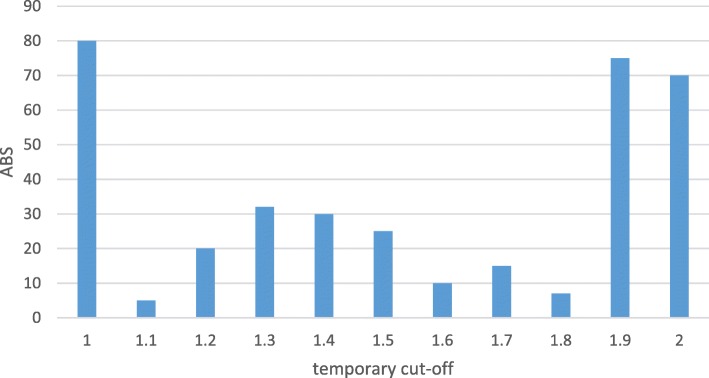
Area Between Survival curves (ABS) for each temporary cutoff value

Figure 5 shows the ABS results from all temporary cutoffs *c* for the exemplary numeric values 1.0 to 2.0 of a fictitious, but typical numeric attribute.

Here, ABS peaks at the lower and upper ends of the temporary cutoffs can be observed. The reason for such extremes is that cutoffs resulting in groups with a very low number of cases show a tendency to survival functions with an extreme step shape. For this reason, we create a Weighting Function (WF) as a second step, in order to smooth the results from the first step:

7 $$ \mathrm{WF}\left(\mathrm{cutoff}=c\right)={\left(p\cdotp \left(1-p\right)\right)}^q $$

**Fig. 6:**
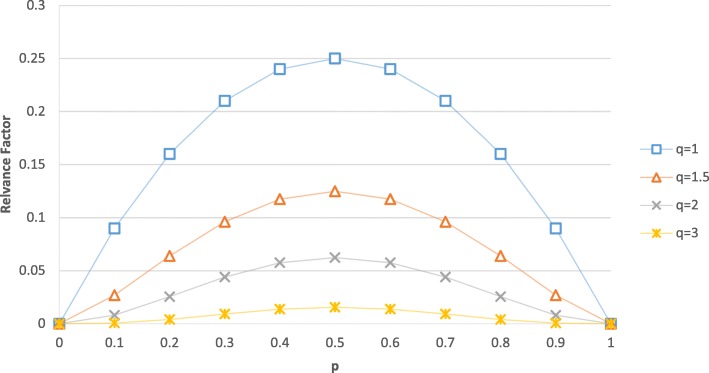
Effect of different smoothing factors

A smoothing factor can be configured with the variable q in Eq. (7). With q = 0 no smoothing effect is taking place. Smoothing factor q ≥ 1 will punish the critical cutoff values on the lower and upper cutoff range, while the midrange cutoff values are in favor (Fig. 6).

The variable *p* is the ratio between the number of cases with a value less or equal to the temporary cutoff *c* and the total number of cases with any numeric value in the numeric attribute:

8 $$ p\left(\mathrm{cutoff}=c\right)=\frac{\mathrm{number}\ \mathrm{of}\ \mathrm{cases}\ \left(\mathrm{numeric}\ \mathrm{value}\le \mathrm{cutoff}\ \right)}{\mathrm{number}\ \mathrm{of}\ \mathrm{cases}\ \left(\mathrm{numeric}\ \mathrm{value}\mathrm{s}\ \mathrm{available}\right)} $$

**Fig. 7:**
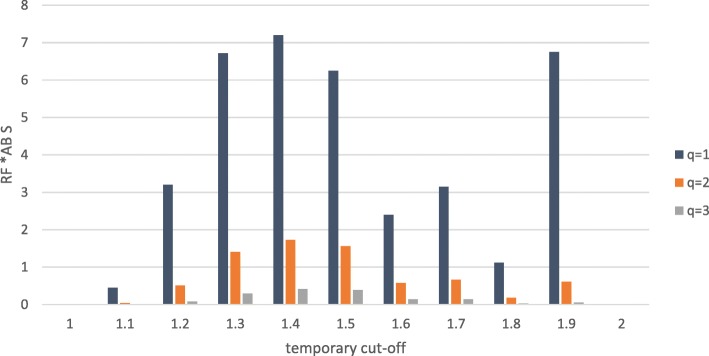
ABS weighted with Weighting Function (WF)

The final cutoff value for the nominalization will now be chosen from the temporary cutoff point with the maximum weighted ABS (cf. Figure 7). This dichotomized version of the numeric attribute will now be handled like any other regular nominal attribute.

#### Global similarity

For a global similarity measure, we suggest to apply an approved global similarity calculation between two cases *c* and *c*^∗^: the Euclidean distance. Empty or other unknown attribute values are, like in many other similarity measures, considered as equal to any other value of a given attribute.

The local similarity between nominal attributes is determined with the method in section 2.3.1, for numeric attributes, the methods from 2.3.3 are used. A common extension is the embedding of a weight factor ${\omega}_{A_i}$ for an attribute *A*_*i*_ with *i* = {1, 2, …, *n*} to emphasize or mitigate the contribution effect of each attribute. However, it is important to know that our method for weight calculation might not be suitable for use in conjunction with our local similarity method, because both approaches are based on the attribute’s survival time and thus, would have impact twice. Instead, it can be used independently for other similarity measures as an alternative weight calculation or feature selection method. Thus, our proposed similarity measure ${\omega}_{A_i}$ can be used for manual fine-tuning or simply be left with the value of 1. The resulting similarity measure is put together in eqs. (9) and (10).

9 $$ {sim}_{global}\left(T|c,{c}^{\ast}\right)=\sqrt{\sum \limits_{i=1}^n{\left({\omega}_{A_i}\bullet {\mathrm{sim}}_{local}\left(T|{a}_i,{a_i}^{\ast}\right)\right)}^2} $$

10 $$ {\mathrm{sim}}_{local}\left(T|{a}_i,{a_i}^{\ast}\right)=\left\{\begin{array}{c}1,\kern0.5em if\ {a}_i\  or\ {{\mathrm{a}}_i}^{\ast }\  is\ unknown, else\\ {}\exp \left(-\left|\mathrm{ABS}\left(T|{a}_i,{a_i}^{\ast}\right)\right|\right), if\ {a}_i\  is\ nominal, else\\ {}\exp \left(-\left|\mathrm{ABS}\left(T|{a}_i\le c,{a}_i>c\right)\right|\right)\end{array}\right. $$

## Results

In the following two sections, we describe aspects to be considered when implementing the similarity measure. They include preprocessing steps for the data used for cases descriptions and workflow for the similarity measure. The evaluation section covers capability aspects using in silico datasets and compares results with competing similarity measures.

### Implementation

#### Preprocessing

The missing link in the processing chain of the usage of the similarity measure in a real clinical domain concerns the preprocessing of input data for the case base. Depending on the state of the input data, different steps might have to be applied before clinical data can be used. Typically, these steps include cleaning, validating, and restructuring data if necessary. The following list gives a basic overview of preprocessing steps that we consider especially relevant for clinical data:

- Spelling correction
- Checking of values for completeness
- Filtering of attributes and values that are used only for comments
- Harmonization/aggregation of values with the same meaning
- Plausibility checks (e.g. numeric attributes may not contain characters, “null” or “unknown”)

Based on our experience, we suggest to eliminate attributes that are only available in few clinical cases. The reason is that a single survival function with only few data points (events) leads to a rough step curve and, in our experience, leads to imprecise and thus unreliable results. Defining a threshold for a minimum number of data items may help to prevent step curved survival functions.

#### Workflow of the similarity measure

**Fig. 8:**
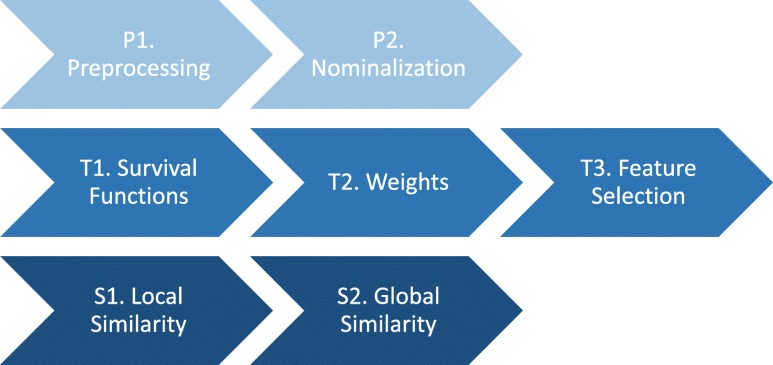
Workflow to compare two cases for similarity

In the previous chapter, we described all necessary steps to calculate the overall similarity of two cases with any available structured clinical data. The following workflow (illustrated in Fig. 8) is summarizing these steps and putting them into order for implementation. It furthermore distinguishes between the steps used for the preparation (P1 and P2), training of the similarity measure (T1-T3) and for the application of the similarity measure for the retrieval (S1 and S2).

P1. Preprocessing: data cleaning, aggregation, remapping and plausibility checks.

P2. Nominalization: transformation of numeric attributes into distinguishable nominal values.

T1. Survival Functions: calculation of the survival function for each value of every attribute.

T2. Weights: calculation of the weight for every attribute.

T3. Feature selection: identification of attributes with high impact on survival.

S1. Local similarity: application of the local similarity algorithm for each attribute with high survival impact.

S2. Global similarity: application of the global similarity algorithm to determine the overall similarity using the attribute weights from workflow step T2.

### Evaluation

#### Material

An evaluation case base for similarity measures needs some predefined and clear biomarkers, so that the similarity measure can prove that it is able to detect and quantify the biomarkers’ impact. Also, several case bases with horizontal (number of attributes) and vertical (number of cases) scalability are mandatory for extensive testing of our approach. Since a clinical data set fulfilling these criteria with sufficient quality is hardly accessible, we decided to design a data set in silico. For this purpose, we implemented a survival data set generator called “vivaGen”, which enables the creation of custom case bases with adjustable survival behavior of each single attribute as well as the overall survival [33]. The program code of “vivaGen” is open source and publicly available [34].

For this evaluation, we used “vivaGen” to generate a set of ten random case bases with an identical base configuration to simulate data from a trial. Each case base consists of *n* = 1000 cases, where each case is described by a total number of 28 attributes: 24 with random values and four special attributes to simulate biomarkers. The outcome attributes in the generated data sets consist of survival time, survival status, and therapy arm (arm A and B). In order to reduce the complexity for this evaluation, both arms perform equally well in terms of overall survival time. The random attributes are created with the help of common distribution functions, namely the normal, exponential, Weibull and the uniform distribution. Especially important, however, are the biomarker attributes because they are generated with a significant impact on the survival time of a case when a biomarker’s value approaches a defined value. For example, the numeric biomarker for arm A has two extreme value ranges: if the value is around 120 the biomarker is defined as being “present” and the case’s survival time is significant higher than average. On the contrary, cases with values around 80 will receive a random survival time, like the random non-biomarker attributes. The nominal biomarkers in “vivaGen” are created with the help of the binomial distribution and internal configuration variables to discriminate between short- and long-time survivors (STS and LTS). Further details about the parametrizing can be obtained from the Additional file 1.

#### Preparation

In the following two sections we will introduce scenarios to evaluate our similarity methods. For each scenario the identical set of case bases from “vivaGen” are used. The generated data sets have no missing values or other undefined attribute values and the feature selection step (T3) is not necessary for such designed data. For discretization of numeric values, we applied a value of 2 for the parameter q.

#### Biomarker detection

In the following evaluation scenario the accuracy of the biomarker matching of similar cases is evaluated. Our basic assumption is that similar cases are expected to have a high matching rate in attributes with a high impact on survival time, which is the case for the biomarker attributes in the generated data set.

In order to receive similarity values between complete cases we applied our suggested global similarity measure from section 2.3.4. For each of the generated case bases we performed a leave-one-out cross-validation with inclusion of the k = 10 (i.e. 1%) most similar cases as results.^1^ It should be mentioned that we decided to place back each test case after drawing (urn model) in order to prevent an increasing instability of the global similarities results due to a running out of remaining training cases.

To see how our similarity measure performs in comparison with others, we considered several similarity measures as potential counterparts. The main criteria for choosing similarity measures for comparison was the capability of working on our datatypes and data structures. For example, textual similarity measures were dismissed because in our context of survival data we do not have textual information. Consequently, we selected the similarity measures HEOM and DVDM (introduced in Section 1.2) and, in addition, a “random pick” algorithm to show how they performed on the generated data sets in each situation. Results are available in Table 1.

**Table 1:** Statistical values for biomarker detection over 10 data set iterations. Our proposed survival-time-based similarity measure (STSM) is compared to the Heterogeneous Euclidian-Overlap Metric (HEOM), Discretized Value Difference Metric (DVDM) and a random pick algorithm

	Numeric Biomarker for arm A				Nominal Biomarker for arm A			
	Mean accuracy (SD)	Mean precision (SD)	Mean recall (SD)	Mean F1-score (SD)	Mean accuracy (SD)	Mean precision (SD)	Mean recall (SD)	Mean F1-score (SD)
STSM	0,944 (0,043)	0,946 (0,044)	0,946 (0,044)	0,946 (0,044)	0,998 (0,002)	0,999 (0,001)	0,993 (0,006)	0,996 (0,004)
HEOM	0,657 (0,013)	0,678 (0,029)	0,684 (0,032)	0,681 (0,03)	0,831 (0,004)	0,759 (0,011)	0,638 (0,013)	0,694 (0,012)
DVDM	0,564 (0,064)	0,595 (0,057)	0,596 (0,058)	0,596 (0,057)	0,644 (0,046)	0,401 (0,081)	0,37 (0,06)	0,384 (0,07)
RANDOM	0,502 (0,007)	0,536 (0,034)	0,535 (0,034)	0,535 (0,034)	0,582 (0,01)	0,3 (0,01)	0,298 (0,011)	0,299 (0,01)
	**Numeric Biomarker for arm B**				**Nominal Biomarker for arm B**			
	**Mean accuracy (SD)**	**Mean precision (SD)**	**Mean recall (SD)**	**Mean F1-score (SD)**	**Mean accuracy (SD)**	**Mean precision (SD)**	**Mean recall (SD)**	**Mean** **F1-score** **(SD)**
STSM	0,909 (0,05)	0,914 (0,048)	0,915 (0,048)	0,915 (0,048)	0,997 (0,003)	1 (0)	0,99 (0,009)	0,995 (0,005)
HEOM	0,661 (0,012)	0,685 (0,025)	0,7 (0,019)	0,692 (0,022)	0,83 (0,003)	0,76 (0,009)	0,648 (0,022)	0,699 (0,016)
DVDM	0,535 (0,012)	0,573 (0,022)	0,577 (0,032)	0,575 (0,025)	0,671 (0,105)	0,467 (0,188)	0,424 (0,151)	0,444 (0,168)
RANDOM	0,505 (0,009)	0,546 (0,028)	0,545 (0,03)	0,546 (0,029)	0,574 (0,013)	0,303 (0,013)	0,303 (0,014)	0,303 (0,013)

Over all iterations, we measured a mean accuracy rate of matching biomarkers for our survival-time-based similarity measure (STSM) between 0.909 (numeric biomarker for arm B) and 0.998 (nominal biomarker for arm A). The HEOM performed with a lower accuracy between 0.657 and 0.831. As expected, the DVDM approach does not perform well with a randomized outcome (here: “therapy arm”) in the training data set. The biomarker matching accuracy of 0.535–0.671 is hardly higher than a similarity measure that randomly picks cases (around 0.5).

#### Determine the weights of attributes

The determination of the weights of attributes is one of the steps in the training phase of the similarity measure (T2) that has essential impact on the subsequent feature selection step. Table 2 shows the calculated weights for each of the ten iterations (IT) of the evaluation data sets. For a better readability the weight values are multiplied with factor 10, which does not affect the results. The average weight of the random attributes has a value of 0.492, which is roughly half the size of the average weight over all attributes (0.897). On the contrary, the weights of biomarker attributes are in the range between 3.242 (nominal biomarker for arm B) and 3.421 (numeric biomarker for arm B). This means a roughly 3.7 [3.61–3.81] times higher weight value than the average over all attributes, and even 6.8 [6.59–6.95] times higher weight than non-biomarker attributes.

**Table 2:** Calculated weights over all attributes, only non-biomarker, and biomarker attributes over ten random case bases. The weight values are scaled by factor 10. Additionally, the relative weight difference to the average weight over all attributes is given

	All attributes	Non-biomarkers		Num. biomarker arm A		Nom. biomarker arm A		Num. biomarker arm B		Nom. biomarker arm B	
	Avg. Weight	Avg. Weight	Rel. (%)	Weight	Rel. (%)	Weight	Rel. (%)	Weight	Rel. (%)	Weight	Rel. (%)
IT#1	0.940	0.504	−46	3.609	+ 284	3.636	+ 287	3.875	+ 312	3.102	+ 230
IT#2	0.791	0.418	−47	2.950	+ 273	2.689	+ 240	3.018	+ 281	3.469	+ 338
IT#3	0.929	0.548	−41	3.028	+ 226	3.035	+ 227	3.416	+ 268	3.382	+ 264
IT#4	0.819	0.435	−47	3.287	+ 301	3.219	+ 293	2.962	+ 262	3.028	+ 270
IT#5	0.852	0.441	−48	3.445	+ 304	3.354	+ 294	3.652	+ 329	2.827	+ 232
IT#6	0.903	0.459	−49	3.432	+ 280	4.109	+ 355	3.368	+ 273	3.354	+ 271
IT#7	1.020	0.622	−39	3.145	+ 208	3.185	+ 212	3.587	+ 252	3.712	+ 264
IT#8	0.871	0.481	−45	3.145	+ 261	3.238	+ 272	3.500	+ 302	2.972	+ 241
IT#9	0.951	0.547	−42	3.386	+ 256	3.593	+ 278	3.599	+ 279	2.912	+ 206
IT#10	0.898	0.466	−48	3.753	+ 318	3.315	+ 269	3.233	+ 260	3.658	+ 307
Mean	0.897	0.492	−45	3.318	+ 271	3.337	+ 273	3.421	+ 282	3.242	+ 262
SD	0.064	0.060	–	0.242	–	0.362	–	0.271	–	0.300	–

In our approach, the purpose of the calculations of weights for attributes is to detect survival time differences. As mentioned above, the biomarker attributes in the generated data sets are designed to have a significant impact on survival time. In this sense, the results indicate the expected behavior.

## Discussion

In this paper, we introduced modular methods for creating a similarity measure by defining similarity on the basis of survival data of attribute values. Where reasonable, each method may be combined with the others to form a similarity measure, as we did. It is also possible to substitute parts of an already established measure with our methods, for example the weight calculation or feature selection.

In clinical domains with limited knowledge about the best possible therapy, like in clinical trials, our approach brings its strength into play. To support finding the hardly predictable “best suitable” therapy for a new patient, it makes sense to take more than just the most similar case into consideration for a therapy decision. The reason is that the applied therapy in the retrieved similar cases still may not necessarily be the individually best. However, a collection of similar patients is especially valuable because of the documented outcomes, which clinicians can now further analyze. They may decide to choose the same therapy for a new patient if the treatment worked well for one or more of the most similar patients or choose a different therapy if it did not perform well in the past. A cancer disease of an individual can be diagnosed in different granularities, because a huge number of specific subtypes was found for many cancer types [35, 36] and probably many more are not known yet. A similarity measure that derives similarity from survival time calculates the survival impact of each attribute in order to determine similarity. However, it is often not clear, if a parameter that could possibly act as a biomarker for subtypes is measured at all. Likewise, biologic effects might have more complex interactions between several causes that might be measured by attributes or not. Such dependent effects are currently not covered by the method presented here. This also affects the case of rare subtypes: If a subtype that is only expressed by a small subset of the population, it is reflected by our similarity measure if it is associated to a parameter value of a single attribute and has significant impact on survival. Any subtype that requires a combination of attributes is currently not addressed by this approach. However, if such a combination is known a priori, it might be possible to use this knowledge to adjust weighting factors if appropriate.

Another effect with impact on the similarity calculations might occur if survival data were acquired in clinical trials: Since trials typically have rigorous inclusion criteria this might lead to a bias in the distribution of the attribute values if they are not independent from the inclusion criteria. For example, the value range may be tighter than expected as compared to that observed in the general population.

An important subject in general data analysis is the handling of missing values. Unlike our in silico data sets for evaluation purposes, actual clinical routine or trial data might include incomplete documentation with missing or mistyped values. Leaving out cases with only few missing values may not be effective and can dramatically decrease the number of cases, which is especially relevant for rare diseases with a low number of cases. For optimal performance a high number of cases and gap-less data sets are desirable. To tackle this issue, for example the maximum likelihood estimation [37] or the expectation–maximization algorithm [38] could be applied to interpolate missing values.

Our method of using survival functions for patient comparisons is a first experiment for a similarity measure of this kind. It is conceivable that this basic approach is adaptable in domains with different assessments of therapy success [39]. For example, in palliative medicine, progression-free survival is often the secondary endpoint after the overall survival [40]. In trials of the domains gynecology and neonatology the days before planned delivery is considered a therapy-deciding outcome [41] and in radiotherapy this is the case for the re-bleeding-free survival [42].

In the evaluation section we showed that our methods behave as expected on our in silico data sets: the detection rate of the artificial biomarkers is much higher than other competing similarity metrics and also the weighting function results correctly in higher values for biomarker than non-biomarker attributes. As a next step, we are working on a validation concept with patient data from electronic health records or clinical trials. The main issue here is that for many malignant diseases therapy options are very limited, or in case of clinical trials, may not be allowed due to the study protocol. Clinical data sets of a cancer disease with nowadays well-proven risk factors might be helpful, if they contain both, cases with ineffective obsolete as well as effective modern therapies. In this case, the calculated weights of the risk attributes for the effective therapies should be significantly higher than those of the obsolete therapies. As a first evaluation on an actual clinical data set we have the “colon” data set [43] in mind, available in the R package “survival” [44].

## Conclusions

In silico evaluation scenarios showed that the mean accuracy of biomarker detection is higher than for competing similarity measures, such as HEOM or DVDM. The weight calculation method showed a more than six times higher weight for biomarker attributes over non-biomarker attributes. These results suggest that the similarity measure described here is suitable for applications based on survival data.

## Supplementary information

**Additional file 1.** Parameters for data set generator tool “vivaGen”.

## Data Availability

We extended the existing Java-based CBR framework myCBR in the version 3.1 to implement the survival function analysis as well as the similarity measure, like proposed in the workflow of Section 5.2. On top of this backend we implemented a graphical user interface named “myCBRBuilder” for visualization of survival functions and performing specific similarity retrievals. The source code is published with an open source license and is freely available [45].

## Abbreviations

ABS: Area between survival functions
CBR: Case-based reasoning
DVDM: Discretized value difference metric (VDM)
HEOM: Heterogeneous Euclidian-overlap metric
IVDM: Interpolated value difference metric (VDM)
LTS: Long-time survivor
OS: Overall survival
Q-CBR: Qualitative case-based reasoning
QSR: Qualitative spatial reasoning
STS: Short-time survivors
STSM: Survival-time-based similarity measure
VDM: Value difference metric
WF: Weighting function
WVDM: Windowed value difference metric (VDM)

## Footnotes

1 In total, this leads to 100.000 single results considered for biomarker classification.
