# Supplementary material for: Methods for a similarity measure for clinical attributes based on survival data analysis
Source: BMC Med Inform Decis Mak. 2019 Oct 21;19:195. doi: 10.1186/s12911-019-0917-6 (PMC6805472; doi:10.1186/s12911-019-0917-6)
Supplement: Supplementary file 1 — Additional file 1. Parameters for data set generator tool “vivaGen”. [file 12911_2019_917_MOESM1_ESM.docx]

## Parameters for data set generator tool “vivaGen”

|  |  | Distribution | Settings | Type | Amount |
| --- | --- | --- | --- | --- | --- |
| Random  $X\sim\mathcal{U}(0,1)\epsilon\left[ 0,1 \right]$ | | Normal | $\mathcal{N}\left( \mu,\sigma^{2} \right), \mu=10\cdot X, \sigma=10\cdot X$ | Numeric | 3 |
|  |  | Exponential | $\mathrm{Exp} \left( \lambda\right), \lambda=10\cdot X$ | Numeric | 3 |
|  |  | Weibull | $\mathrm{Weibull}\left( \lambda,k \right),\lambda=10\cdot X,k=3\cdot X$ | Numeric | 3 |
|  |  | Uniform | $X$ | Numeric | 3 |
|  |  | Uniform | $5\cdot\left\lceil X \right\rceil$  ⇒ maximum 5 unique labels | Nominal | 12 |
| Biomarker | Arm A | Normal | Biomarker present: $\mathcal{N}\left( \mu,\sigma^{2} \right), \mu=120, \sigma=10$  Biomarker not present: $\mathcal{N}\left( \mu,\sigma^{2} \right), \mu=80, \sigma=7$ | Numeric | 1 |
|  |  | Binomial | p(survivor\|LTS)=0.77 p(non-survivor\|LTS)=0.1 p(STS)=0.1 | Nominal | 1 |
|  | Arm B | Normal | Biomarker present: $\mathcal{N}\left( \mu,\sigma^{2} \right), \mu=20, \sigma=4$  Biomarkern not present: $\mathcal{N}\left( \mu,\sigma^{2} \right), \mu=40, \sigma=4$ | Numeric | 1 |
|  |  | Binomial | p(survivor\|LTS)=0.77 p(non-survivor\|LTS)=0.1 p(STS)=0.1 | Nominal | 1 |
